# Antiatopic Dermatitis Effect of *Artemisia iwayomogi* in Dust Mice Extract-Sensitized Nc/Nga Mice

**DOI:** 10.1155/2014/673286

**Published:** 2014-02-23

**Authors:** Hyekyung Ha, Hoyoung Lee, Chang-Seob Seo, Hye-Sun Lim, Mee-Young Lee, Jun Kyoung Lee, Hyeunkyoo Shin

**Affiliations:** ^1^Herbal Medicine Formulation Research Group, Korea Institute of Oriental Medicine, 1672 Yuseongdae-ro, Yuseong-gu, Daejeon 305-811, Republic of Korea; ^2^KM Health Technology Research Group, Korea Institute of Oriental Medicine, 1672 Yuseongdae-ro, Yuseong-gu, Daejeon 305-811, Republic of Korea

## Abstract

*Aims*. *Artemisia iwayomogi* (AI) has been used for fever reduction, diuresis, and hepatoprotection in Korea. The present study was performed to evaluate the anti-inflammatory and antiatopic dermatitis effects of AI using both *in vitro* and *in vivo* systems. *Methods*. The compositions in AI were analyzed by HPLC. To determine the anti-inflammatory effects of AI, the production of nitric oxide (NO) was measured in lipopolysaccharide treated RAW264.7 cells. Histamine levels were assayed to evaluate the antiallergic effects on MC/9 cells stimulated with phorbol-12 myristate 13-acetate and A23187. Finally, AI (10 mg/mouse/day) was topically applied onto the backs and ears of *Dermatophagoides farinae*-sensitized Nc/Nga mice for four weeks. *Results*. Isochlorogenic acid A (20.63 ± 0.26 mg/g), chlorogenic acid (9.04 ± 0.08 mg/g), and scopoletin (8.23 ± 0.01 mg/g) were among the major components of AI. AI inhibited the NO and histamine productions in RAW264.7 and MC/9 cells, respectively. In the mice, the topical application of AI reduced the dermatitis scores in the dorsal skin and ears and reduced the plasma levels of IgE. *Conclusions*. These results suggest that AI might be explored as a potential therapeutic agent to treat AD, and that the analytic method using HPLC will facilitate the development of quality control for AI.

## 1. Introduction

Traditionally, herbal medicine has been used to treat or prevent diseases. *Artemisia iwayomogi* (AI, Compositae) has been used in Korea to treat various diseases, including hepatic failure and inflammatory and immune-related diseases. AI has been known to inhibit hepatic fibrogenesis by reducing oxidative stress and lipid peroxidation [1] and preventing high fat diet- (HFD-) induced obesity and metabolic disorders through the downregulation of gene expressions related to adipogenesis and inflammation in visceral adipose tissues [2]. *A. capillaries* (AC, Compositae), one of the *Artemisia* herbs, has been traditionally used as an herbal medicine in East Asia and has been reported in pharmacopoeias in Korea, China, and Japan. However, AI is only mentioned in the Korean herbal pharmacopoeia. The efficacies of AI have been less reported than those of AC because AI is not traditionally used in other countries.

Atopic dermatitis (AD) is a common inflammatory, chronic or chronically relapsing, noncontagious, and pruritic skin disorder [3]. AD is often accompanied with allergic inflammation, which is initiated by the activation of the adaptive immune response. Immunoglobulin E (IgE) is produced in plasma cells and bound by mast cells in type I allergic reactions. The IgE-primed mast cells release chemical mediators, such as histamine, leukotrienes (LTs), and prostaglandin D_2_ (PGD_2_). These mediators lead to immediate phase reactions in the tissue, such as redness and itching, shortly after allergen-IgE binding. In the later phases of the disease, cytokines (IL-4 and IL-13) and chemokines are generated and released several hours after allergen-antibody cross-linking [4].

Topical corticosteroids (TCs) and nonpharmacological treatments, such as emollients, are first-line prescription therapies for AD. TCs are currently the most potent treatment for AD. However, their chronic use can also be associated with significant adverse effects, such as the thinning of the skin, adrenal gland suppression, and treatment resistance [5, 6]. As a second-line therapy, topical calcineurin inhibitors (TCIs), such as tacrolimus and pimecrolimus, have been reported to be effective for atopic dermatitis. TCIs have been developed to suppress the results of immune responses during rejection in organ transplant recipients. However, concerns over systemic toxicity have limited their use. Topical tacrolimus ointment causes transient burning in ~60% of patients [7, 8]. Subsequently, the need to efficiently manage the AD response while reducing its side effects has led to the development of alternative remedies.

AI has been used to treat allergic diseases; the effects of AI on atopic dermatitis (AD), an inflammatory allergic disease, have not been established to date. The present study was conducted to analyze the contents of the ingredients in AI and to evaluate the anti-inflammatory and antiallergic effects of AI in lipopolysaccharide- (LPS-) treated RAW264.7 cells and phorbol-12 myristate 13-acetate (PMA) and A23187-treated MC/9 cells, respectively. In addition, AI was applied to analyze these treatments' effects on the AD response in Nc/Nga mice.

## 2. Materials and Methods

### 2.1. Chemicals and Reagents for HPLC Analysis

Chlorogenic acid and caffeic acid were purchased from Acros Organics (Pittsburgh, PA, USA). Scopoletin was purchased from Wako Pure Chemical Industries, Ltd. (Osaka, Japan). Isoquercitrin, isochlorogenic acid A, and isorhamnetin were from Biopurify Phytochemicals Ltd. (Chengdu, China). The purity of the six compounds was determined to be ≥98% by HPLC analysis. HPLC-grade reagents, methanol, acetonitrile, and water were obtained from J.T.Baker (Phillipsburg, NJ, USA). Glacial acetic acid was of analytical reagent grade and was procured from Junsei (Tokyo, Japan).

### 2.2. HPLC Conditions

HPLC analysis was performed using a Shimadzu LC-20A HPLC system (Shimadzu Co., Kyoto, Japan) consisting of an LC-20AT pump, DGU-20A_3_ online degasser, SPD-M20A detector, SIL-20AC autosampler, and CTO-20A column oven. The data processor used was LC Solution software (version 1.24, Shimadzu Co., Kyoto, Japan). The column for separation used was a Gemini C_18_ (250 × 4.6 mm; particle size 5 *μ*m, Phenomenex; Torrance, CA, USA). The mobile phases were composed of A (1.0% aqueous acetic acid, v/v) and B (acetonitrile with 1.0% acetic acid, v/v). The gradient flow was as follows: 0–5 min, 10–10% B; 5–30 min, 10–50% B; 30–35 min, 50–50% B; 35–40 min, 50–10% B. The temperature of the column oven was maintained at 40°C. The analysis was performed at a flow-rate of 1.0 mL/min with PDA detection at 254, 320, and 340 nm. The injection volume was 10 *μ*L.

**Figure 1:**
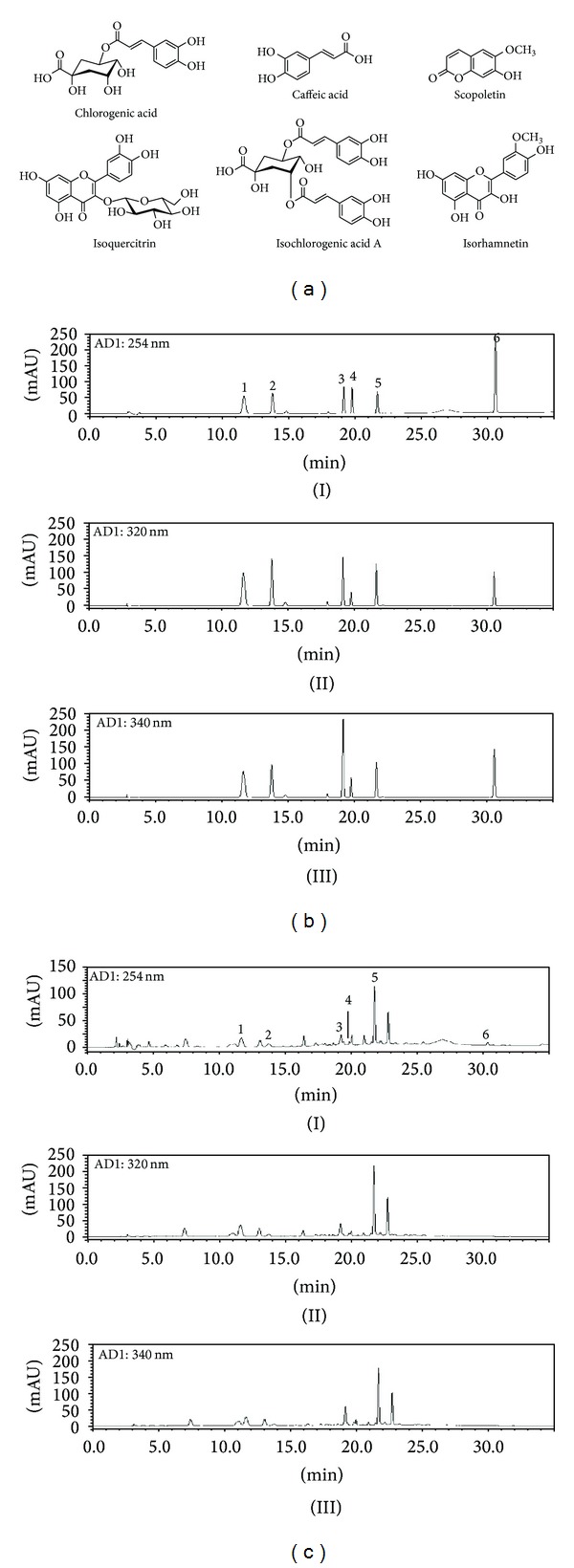
Chemical structures of the ingredients of *A. iwayomogi* (AI; A) and HPLC chromatograms of reference standards (b) and AI extracts (c). The detection wavelengths were 254 nm (I), 320 nm (II), and 340 nm (III). Chlorogenic acid (1), caffeic acid (2), scopoletin (3), isoquercitrin (4), isochlorogenic acid A (5), and isorhamnetin (6).

Standard stock solutions of six compounds, chlorogenic acid, caffeic acid, scopoletin, isoquercitrin, isochlorogenic acid A, and isorhamnetin (Figure 1) were dissolved in methanol at concentrations of 1.0 mg/mL and kept below 4°C. Working standard solutions were diluted to calibration curves in the concentration range of 0.78–50.00 *μ*g/mL for chlorogenic acid and scopoletin, 0.16–10.00 *μ*g/mL for caffeic acid, isoquercitrin, and isorhamnetin, and 2.34–150.00 *μ*g/mL for isochlorogenic acid A, respectively.

### 2.3. Preparation of Water Extract and Sample Solution

The dried AI (2.5 kg) was extracted with water (25 L × 1 time) at 100°C for 2 h using an herb extractor (COSMOS-660, Kyungseo Machine Co., Inchon, Korea). AI was purchased from HMAX (Jecheon, Korea) in June 2008. These materials were confirmed taxonomically by Professor Je-Hyun Lee of Dongguk University, Korea. A voucher specimen (AI-2008-ST27) was deposited at the Herbal Medicine Formulation Research Group, Korea Institute of Oriental Medicine. The extract solution was filtered through filter paper and freeze-dried (359.51 g). The yield of extract was 14.38%. The lyophilized extract (20 mg) was dissolved in 70% ethanol (10 mL) and then filtered through a 0.2 *μ*m membrane filter (Woongki Science, Seoul, Korea) before being injected into the HPLC for simultaneous determination.

### 2.4. Nitric Oxide (NO) and PGE_2_ Productions in LPS-Stimulated RAW264.7 Cells

The murin macrophage cell line, RAW264.7, was obtained from the American Type Culture Collection (ATCC, Rockville, MD, USA) and cultured in Dulbecco's modified Eagle's medium (DMEM, Gibco BRL., NY, USA) containing 5.5% (v/v) heat-inactivated fetal bovine serum (FBS, Gibco BRL., NY, USA) and 100 U/mL penicillin and 100 *μ*g/mL streptomycin (Gibco BRL, NY, US). The cells were seeded in 96-well plates (2.5 × 10^3^ cells/well) for the cytotoxicity assay and then incubated with various concentrations of AI extract (10, 50, and 100 *μ*g/mL) for 24 hr. The final volume of culture medium was 100 *μ*L/well. The vehicle control was 1% DMSO. After treatment, 10 *μ*L of Cell Counting Kit-8 reagent (CCK-8, Dojindo, Japan) was added to each well and the plates were incubated for 4 hr. The absorbance was measured at 450 nm using a microplate reader (Benchmark Plus, Bio-Rad Laboratories Inc., PA, USA), and the percentages of viable cells were calculated. Cytotoxicity did not occur on RAW264.7 cells after the treatment of the AI extract in the range of 10–100 *μ*g/mL.

For the NO and PGE_2_ assays, RAW264.7 cells were seeded at a density of 5 × 10^5^ cells/well in the 48-well plates. After 16 hr, the cells were stimulated with 1 *μ*g/mL of LPS in the absence or presence of AI (10–100 *μ*g/mL) for 18 hr. *N* ^*G*^-methyl-L-arginine (NMMA; Sigma-Aldrich, Inc., MO, USA) and indomethacin (Sigma-Aldrich, Inc.) were used as the positive controls to inhibit NO and PGE_2_ production, respectively. The final volume was 500 *μ*L/well. The samples were treated with LPS on the cells at the same time. After incubation, the cell culture media were collected and used to measure the NO (A Griess reagent system, Promega., WI, USA) [9] and PGE_2 _(PGE_2 _ELISA kit, Cayman Chemical Co., MI, USA) levels according to the manufacturer's protocols.

### 2.5. Histamine Production in PMA and A23187-Stimulated MC/9 Cells

The rat mast cell line, MC/9, was obtained from ATCC and cultured in Iscove's modified Dulbecco's medium (Gibco BRL., NY, USA) before being supplemented with 10% heat-inactivated FBS, penicillin (100 U/mL), and streptomycin (100 *μ*g/mL) in a 5% CO_2_ incubator at 37°C. The cytotoxicity for AI was assayed with the same method as in RAW264.7 cells. There was no cytotoxicity in the MC/9 cells after treatment with the AI extracts at concentrations below 50 *μ*g/mL. For the histamine production assay, the cells were seeded in 48-well plates (2 × 10^5^ cells/well) and cultured for 16 hr. After incubation, MC/9 cells were stimulated with phorbol 12-myristate 13-acetate (PMA, 50 nM, Sigma-Aldrich, St Louis, MO, USA) and A23187 (1 *μ*M, calcium ionophore, Sigma-Aldrich) in the absence or presence of AI (25 and 50 *μ*g/mL) for 24 h [10]. The final volume was 500 *μ*L/well. The samples were treated with PMA and A23187 on the cells at the same time. The supernatants were collected, and histamine production was measured with an ELISA kit, according to the protocol provided by Oxford Biomedical Research (Oxford, MI, USA).

### 2.6. Animals and Sensitization

AD-like skin lesions were generated in male NC/Nga mice (Central Laboratory Animal Inc., Seoul, Korea) using *Dermatophagoides farinae* extract ointment (Biostir-AD, Biostir Co., Ltd., Kobe, Japan) [11]. The mice were housed individually in an air-conditioned room maintained at 24 ± 2°C with 55 ± 15% relative humidity. All procedures involving animals were conducted in accordance with the guidelines of the Institutional Animal Care and Use Committee of the Korea Institute of Oriental Medicine (approval number: #10-052).

Ten-week old mice were divided randomly into four groups with seven mice per group: the unsensitized (normal; 200 *μ*L of 70% EtOH/mouse/day), *D. farinae*-sensitized (control; 200 *μ*L of 70% EtOH/mouse/day), *D. farinae*-sensitized plus Protopic ointment-treated (Protopic; 50 mg/mouse/day), and *D. farinae*-sensitized plus AI extract-treated (AI; 10 mg/mouse/day) groups. For sensitization, 50 mg Biostir-AD was topically applied on the upper dorsal skin and the backs of the ears twice weekly for 4 weeks. The AI extract was dissolved in the vehicle solution (70% EtOH). The Protopic ointment was used as a positive control. The vehicle, AI extract, and positive control were applied on the upper dorsal skin and ears every day during the 4 weeks of sensitization.

### 2.7. Dermatitis Score

The dermatitis scores were assessed on the dorsal skin and ears once a week for 4 weeks. The Eczema Area and Severity Index (EASI) scoring system was applied to assess the severity of the dermatitis. This was defined as the sum of the scores for the erythema/hemorrhage, edema, excoriation/erosion, and scaling/dryness as follows: no symptoms, 0; mild, 1; moderate, 2; and severe, 3 [12].

### 2.8. Histological Analysis

After 4 weeks of treatment, the mice were anesthetized by i.p. injections of pentobarbital sodium (Entobar inj., Hanlim Pharm. Co., Ltd., Korea). Blood samples were collected from the inferior *vena cava* and then stored in the Microtainers (Becton, Dickinson and Company, NJ, USA) containing K_2_-EDTA. Plasma samples were collected after centrifugation at 10000 rpm for 10 min and stored at −80°C. A complete gross observation was performed on all terminated animals. The dorsal skin and one ear of each mouse were taken and fixed in 10% (v/v) neutral buffered formalin for 24 hr. The tissues were embedded in paraffin and then sectioned at 4 *μ*m thickness. The tissue sections were stained with hematoxylin and eosin (H and E) to estimate the epidermal inflammation (hypertrophy and infiltration by inflammatory cells). Mast cells of dermis were stained with toluidine blue and the sections were observed under the microscope. Average mast cell numbers were measured by counting 3 different areas in each slide of skin (×100), and the mean number of cells was calculated.

### 2.9. Plasma Levels of IGE and Histamine

The plasma levels of IgE (Bethyl Laboratories Inc., USA) and histamine (Oxford Biomedical Research, USA) were quantified using ELISA kits according to the manufacturer's instructions.

### 2.10. Statistical Analysis

The data were analyzed using a one-way ANOVA followed by the Bonferroni multiple comparison test; all date are expressed as the means ± SEM. A *P* value <0.05 was defined as statistically significant. All statistical analyses were performed using the SYSTAT 8.0 program (SYSTAT Inc., Evanston, IL, USA).

## 3. Results

### 3.1. Quantitation of Ingredients in AI

A chromatogram of AI extract was obtained using an HPLC-PDA. Under optimized chromatography conditions, six compounds were eluted within 35 min in the sample analysis using mobile phases comprising solvent A (1.0%, v/v, acetic acid in water) and solvent B (1.0%, v/v, acetic acid in acetonitrile).

The linearity of the peak area (*y*) versus concentration (*x*,  *μ*g/mL) curve for each component was used to calculate the contents of the main components in the AI extract. The correlation coefficients (*r* ^2^) of the calibration curves for the six main constituents were ≥0.9999. The line equations and *r* ^2^ values for the calibration curves are summarized in Table 1. The retention times of chlorogenic acid, caffeic acid, scopoletin, isoquercitrin, isochlorogenic acid A, and isorhamnetin were 11.63, 14.80, 15.55, 19.78, 21.63, and 30.53, respectively. Figure 1 shows the HPLC chromatogram of standard solution and water extracts of AI. Isochlorogenic acid A was a major ingredient (20.63 ± 0.26 mg/g) in our analytical system, and the contents of the six constituents ranged from 0.64 to 20.63 mg/g. The analytical results for each component identified are summarized in Table 2.

### 3.2. Inhibitory Effects of AI on NO, PGE_2_, and Histamine Production

**Figure 2:**
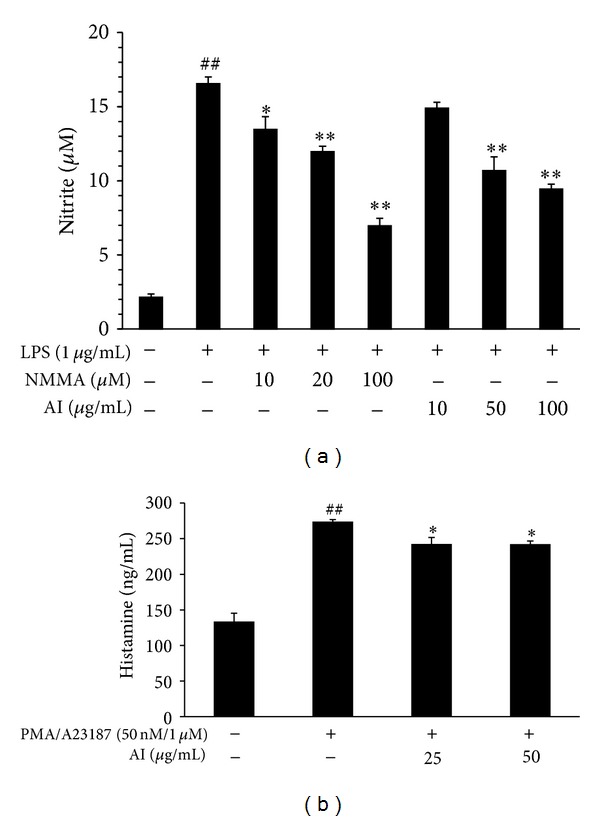
*A. iwayomogi* (AI) extract inhibited NO and histamine production. (a) AI inhibited NO production in LPS-stimulated (1 *μ*g/mL, 24 hr) RAW264.7 cells in a concentration-dependent manner. Each data value represents the mean ± SEM of triplicate experiments (^##^ *P* < 0.01 compared with the control group, *P < 0.05 and **P < 0.01 compared with the LPS-treated group). (b) Histamine production was reduced by AI in PMA- and A23187-treated (50 nM and 1 *μ*M, 24 hr) MC/9 cells. Each data value represents the mean ± SEM of triplicate experiments (^##^ *P* < 0.01 compared with the control group, *P < 0.05 compared with the PA-treated group).

AI was shown to inhibit the inflammatory and allergic responses of *in vitro* systems. LPS-induced NO production was significantly reduced by AI extract in a dose-dependent manner compared to the group treated with LPS alone (*P* < 0.05, Figure 2(a)); however, there was no difference in the PGE_2_ production (data not shown). Histamine production was inhibited by AI in the PMA- and A23187-stimulated MC/9 cells (*P* < 0.05, Figure 2(b)).

### 3.3. Dermatitis Scores in Nc/Nga Mice

**Figure 3:**
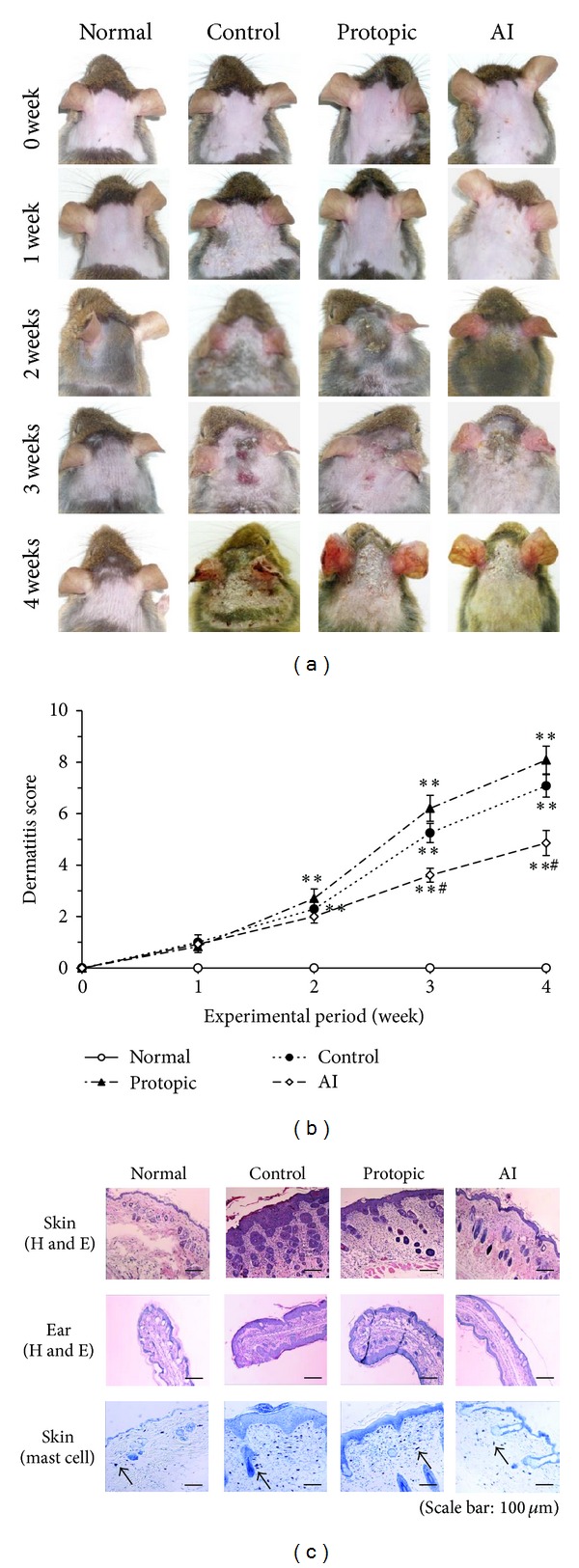
*A. iwayomogi* (AI) extract reduced the dermatitis scores and histological changes in *D. farinae* extract-sensitized Nc/Nga mice. (a) Macroscopic changes following the consecutive administration of AI or Protopic ointment onto *D. farinae*-induced, AD-like lesions on the back and ears in Nc/Nga mice. The images show the backs and ears for four weeks after sensitization. (b) The dermatitis scores of *D. farinae*-induced, AD-like skin lesions on the backs and ears (mean ± SEM (*n* = 5), *P < 0.05 and **P < 0.01, compared with the normal group, ^##^ *P* < 0.01, compared with the *D. farinae*-induced control group). AI (10 mg/mouse/day) and Protopic ointment (50 mg/mouse/day) were topically applied on the back and ears once daily for four weeks. (c) Histological features of the back and ears. The tissues were stained with hematoxylin and eosin (H and E) or toluidine blue to estimate epidermal inflammation or mast cell infiltration. The mast cells are indicated by arrows.

In representative photographs for each group, the mice developed macroscopic lesions on the dorsal skin and ears starting at the second week after the initiation of *D. farinae* extract treatment (Figure 3(a)). The dorsal skin and ear lesion severity of the AI group were significantly reduced compared to those of the control group at the third and fourth weeks (*P* < 0.05). However, the dermatitis scores of the Protopic-treated positive control group were no different than those of the control group. The maximum dermatitis scores were recorded following the fourth week of *D. farinae* extract application in all groups. The scores for each group were as follows: 0.0 ± 0.00 (normal), 7.1 ± 0.43 (control), 8.1 ± 0.54 (Protopic), and 4.9 ± 0.60 (AI) (Figure 3(b)).

### 3.4. Histological Observations

The control group showed significant dorsal skin and ear lesions and hemorrhage, hypertrophy, and hyperkeratosis of the epidermis in the dorsal region (Figure 3(c)). These changes were significantly improved in the AI group; however, there were no changes in the Protopic group. The infiltration of mast cells was reduced by the application of AI in the dorsal skin (Figure 3(c)).

### 3.5. Plasma Levels of Histamine and IGE

**Figure 4:**
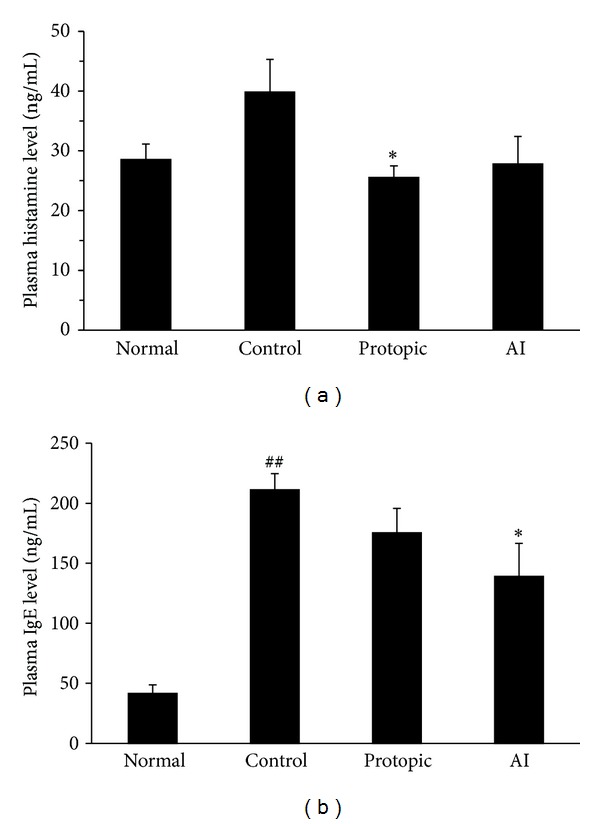
*A. iwayomogi* (AI) extract reduced the plasma levels of histamine (a) and IgE (b) in *D. farinae* extract-sensitized Nc/Nga mice. AI (10 mg/mouse/day) and Protopic ointment (50 mg/mouse/day) were topically applied on the backs and ears once daily for 4 weeks. The concentrations of histamine and IgE were measured by ELISA methods (mean ± SEM, *n* = 5), ^##^ *P* < 0.01 compared with the normal group, *P < 0.05 compared with the *D. farinae*-induced control group.

The plasma histamine levels were elevated by *D. farinae* extract-sensitization (control group, 39.85 ± 5.44 ng/mL) compared to the normal group (28.56 ± 2.56 ng/mL). The histamine levels were reduced in the AI treatment groups (27.83 ± 4.60 ng/mL); however, there was no significant difference compared to the control group (*P* > 0.05). In the Protopic treatment group, the histamine levels (25.54 ± 1.93 ng/mL) were decreased, compared to the control group (*P* < 0.05, Figure 4(a)).

Additionally, the total plasma IgE levels were significantly increased in the control group (211.3 ± 13.4 ng/mL) compared to the normal group (41.56 ± 7.02 ng/mL, *P* < 0.01). AI treatment inhibited increased plasma IgE levels (139.0 ± 27.6 ng/mL) in *D. farinae*-sensitized mice (*P* < 0.01). However, there was no significant difference in the Protopic group (175.4 ± 20.4 ng/mL, Figure 4(b)).

## 4. Discussion

Allergic reactions, including atopic dermatitis (AD), are developed due to an imbalance of Th1 and Th2 cells that is mediated by IgE and histamine levels. AD is characterized by the overexpression of inflammatory cytokines such as IL-10 and by high IgE levels. The involvement of Th2 cells both helps to explain the joint involvement of IgE-producing B cells (via IL-4 and IL-13), mast cells (via IL-4 and IL-10), and eosinophils (via IL-5) in the allergic inflammatory process and accounts for the other pathophysiologic features of allergies [13].

*Artemisia* herbs, including AI, have been used for liver disorders and inflammatory diseases. We investigated the anti-inflammatory and anti-AD effects of AI treatment using *in vitro* and *in vivo *systems because AD is strongly associated with the inflammatory response. AI suppressed the production of NO in LPS-stimulated RAW264.7 macrophage cells. Histamine production was also inhibited by AI in PMA- and A23187-stimulated MC/9 mast cells. Nc/Nga mice are characterized by AD-like skin lesions and elevated levels of blood IgE. The skin changes that developed in the Nc/Nga mice closely mimic human AD, which conventionally develops following infection with mites [14]. The most important allergens associated with human AD are house dust mite allergens, and *D. farinae* is the most common house dust mite present in the environment. The infiltration of mast cells is another important factor in AD development [15]. AI treatment reduced the dermatitis scores after the third week in *D. farinae*-sensitized Nc/Nga mice. In addition, AI suppressed the histological features of the disease, including edema, cornification of the epidermis, and mast cell infiltration in the dorsal skin and ear. Furthermore, treatment with AI reduced the plasma levels of histamine and IgE in *D. farinae* -sensitized Nc/Nga mice.

In the Protopic group, a positive control group, dermatitis scores were not reduced compared with those of control group. As one of the adverse effects of Protopic ointment, the patients experienced skin irritation and burning at the site of application [6]. Therefore, we expected that the high dermatitis scores in the Protopic group would be caused by increased itching behaviors at the application area of the ointment. However, the plasma levels of histamine and IgE were reduced in the mice.

In the present study, the chemical contents of the AI extract were analyzed using a high performance liquid chromatography (HPLC) system. Isochlorogenic acid A, chlorogenic acid, and scopoletin were detected as the major compounds in the AI extract. Isochlorogenic acid A has been reported to have hepatoprotective effects and antioxidative properties through the induction of HO-1 [16] and immunopotentiation properties mediated through the NF-*κ*B-induced release of NO from macrophages [17]. Chlorogenic acid has been reported to inhibit the production of inflammatory mediators and cytokines [18, 19]. Moreover, chlorogenic acid exhibits antibacterial, antioxidant, antihepatic injuries, and antiallergic activities [20, 21]. Lee et al. reported that scopoletin has been shown to suppress the osteoclastogenesis from RAW264.7 cells [22] by scavenging reactive oxygen species (ROS). Scopoletin significantly inhibited interleukin-4 (IL-4), IL-5, and IL-10 production [23]. The antiallergic properties of scopoletin might be mediated by the downregulation of cytokine expression in Th2 cells. Therefore, the analytical method of using HPLC to distinguish the major ingredients of AI, isochlorogenic acid A, chlorogenic acid, and scopoletin, might be a useful quality control method for these AI remedies.

## 5. Conclusions

These results suggest that AI inhibits AD-like skin lesions to reduce the generation of IgE, the inflammatory response on the skin, and the release of preformed mediators, such as histamine, on *D. farinae*-sensitized Nc/Nga mouse model. We conclude that AI should be explored as a potential therapeutic agent to treat allergic diseases, including AD.

## Figures and Tables

**Table 1:** Calibration curves for the six ingredients of *A. iwayomogi *(*n* = 3).

Component	Linear range (*μ*g/mL)	Regression equation^a^	*r* ^2^	LOD (ng/mL)	LOQ (ng/mL)
Chlorogenic acid	0.78–50.00	*Y* = 32627.70*x* − 4523.06	1.0000	126.49	421.62
Caffeic acid	0.16–10.00	*Y* = 60172.54*x* − 1297.07	0.9999	43.64	145.45
Scopoletin	0.78–50.00	*Y* = 34832.43*x* + 3393.40	0.9999	52.00	173.33
Isoquercitrin	0.16–10.00	*Y* = 26489.61*x* + 23.88	1.0000	38.40	128.00
Isochlorogenic acid A	2.34–150.00	*Y* = 38075.16*x* − 21283.50	0.9999	51.62	172.06
Isorhamnetin	0.16–10.00	*Y* = 40367.16*x* − 376.39	1.0000	35.56	118.52

^a^ *Y* represents peak area (mAU); *x* represents concentration (*μ*g/mL).

**Table 2:** Contents of the six ingredients in the 70% ethanol extract of *A. iwayomogi *(*n* = 3).

Compound	Content (mg/g)		
	Mean	SD	RSD (%)
Chlorogenic acid	9.04	0.08	0.89
Caffeic acid	0.96	0.02	1.64
Scopoletin	8.23	0.01	0.12
Isoquercitrin	1.43	0.02	1.21
Isochlorogenic acid A	20.63	0.26	1.27
Isorhamnetin	0.64	0.01	1.46
